# Therapy Targeting Stem Cell in Patients with Decompensated Cirrhosis of Liver in a Tertiary Treatment Care Center of Bangladesh

**DOI:** 10.5005/jp-joumals-10018-1229

**Published:** 2017-05-05

**Authors:** Mamun Al Mahtab, Sheikh MN Alam, Ahmed L Moben, Ruksana Raihan, Mohammad A Alam, Mohammad A Rahim, Mohammad H Uddin, Sheikh Mohammad Fazle Akbar

**Affiliations:** 1 Department of Hepatology, Bangabandhu Sheikh Mujib Medical University, Dhaka, Bangladesh; 2 Department of Medicine, Kurmitola General Hospital, Dhaka, Bangladesh; 3 Department of Virology, Bangabandhu Sheikh Mujib Medical University, Dhaka, Bangladesh; 4 Department of Hepatology, Dhaka Medical College, Dhaka, Bangladesh; 5 Department of Hepatology, Abdul Malek Ukil Medical College, Noakhali, Bangladesh; 6 Clinical Research Organization, Dhaka, Bangladesh; 7 Department of Medical Sciences, Toshiba General Hospital, Tokyo, Japan

**Keywords:** Decompensated liver cirrhosis, Granulocyte colony stimulating factor, Prognosis, Stem cell, Therapy.

## Abstract

**Introduction:**

Decompensated cirrhosis is associated with significantly high mortality resulting from hepatic failure, and liver transplantation seems to be the only viable indication for its management. The objective of this study is to assess if granulocyte colony-stimulating factor (G-CSF), a stimulatory of stem cell *in vivo,* may be of any benefit for patients with decompensated cirrhosis of liver.

**Materials and methods:**

Seventeen consecutive patients with decompensated cirrhosis of liver were recruited in this prospective study. They received injection of G-CSF (30 IU) over a period of 6 weeks (12 injections) in addition to standard of care.

**Results:**

Patients were followed up at the end of treatment and at 12 weeks of treatment. Treatment was well tolerated, and no significant adverse event was recorded in any patient. Fifteen out of 17 (88%) patients were alive at last follow-up. Although serum bilirubin, albumin, and prothrombin time improved in some patients, statistically significant improvement of Child-Pugh score could not be documented.

**Conclusion:**

The study establishes the safety of G-CSF therapy in patients with decompensated cirrhosis of liver. Besides, such therapy may also have survival benefit, although long-term follow-up is needed to assess its real utility in clinical perspectives.

**How to cite this article:** Al Mahtab M, Alam SMN, Moben AL, Raihan R, Alam MA, Rahim MA, Uddin MH, Akbar SMF. Therapy Targeting Stem Cell in Patients with Decompensated Cirrhosis of Liver in a Tertiary Treatment Care Center of Bangladesh. Euroasian J Hepato-Gastroenterol 2017;7(1):113-115.

## INTRODUCTION

Decompensated cirrhosis is characterized significantly by high mortality caused by hepatic failure. Liver transplantation remains the only curative treatment available, but its application is limited by the shortage of living and cadaveric donors and high cost. Moreover, liver transplantation is not available in Bangladesh as of now. Another modality for management of decompensated cirrhosis is liver support device (MARS) that serves as a "bridge" to transplant. However, studies have shown that MARS does not reduce mortality significantly compared with standard medical treatment. In order to overcome all these problems, alternative approaches have been sought for.

In particular, the great potential of stem cells to differentiate into multiple cell lineages raises the exciting hypothesis that these cells can be used in tissue-specific cell regeneration, when tissue-resided stem cells are not sufficient for the regeneration of a failing organ.^1,2^ During liver regeneration, bone marrow-derived hematopoietic stem cells (BMC) may be mobilized to the liver using granulocyte colony stimulating factor (G-CSF) and, together with hepatocytes and intrahepatic stem cells, contribute to the proliferation of liver cells. Therefore, G-CSF therapy may be beneficial for liver regeneration in patients with decompensated cirrhosis of liver.^3,4^

## STUDY DESIGN, RESULTS, AND DISCUSSION

Seventeen consecutive patients presenting with decom-pensated cirrhosis of liver of varied etiology were included in the study (Table 1). The study was conducted at the Department of Hepatology, Bangabandhu Sheikh Mujib Medical University, Dhaka, Bangladesh. Ethical approval for the study was obtained from the Institutional Review Board at the university and informed, written consent was taken from each study participant. Demographic characteristics and etiology of liver cirrhosis are shown in Tables 1 and 2 respectively.

The study establishes safety of G-CSF therapy in patients with advanced liver cirrhosis and sets the platform for further studies, with stem cells adopting different approaches. It did not reveal statistically significant improvement in Child-Pugh score in the study population during the follow-up period. However, improvements in serum bilirubin, albumin, and prothrombin time were recorded in some of the patients included in the study. An important observation was that 88% patients were alive. Two out of 17 patients expired during follow-up. Of them, one died of variceal bleeding on day 24, while the second patient died on day 50 from hepatorenal syndrome (HRS).

There are few papers in the world literature on the use of G-CSF in patients with decompensated cirrhosis of liver. Garg et al^3^ subjected 15 patients with advanced liver cirrhosis (Child-Pugh score > 6 points) to 3-day G-CSF course, administered at 3-month intervals for a total of four courses. Using flow cytometry, BMC mobilization was assessed by evaluating CD34 positive cells. They reported significant increase in G-CSF-induced circulating CD34 positive cells. Treatment was well tolerated. Overall, 10 patients had either improved or had stable liver function tests (such as the Child-Pugh score), whereas five worsened and died from liver-related causes.^1^

**Table 1:** **Table 1:** Etiology of liver cirrhosis in study population

*Etiology*		*Frequency*		*Percent*	
Cryptogenic		*4*		23	
HBV		9		53	
HCV		3		18	
NASH		1		6	
Total		17		100	

HBV: Hepatitis B virus; HCV: Hepatitis C virus; NASH: Nonalcoholic steatohepatitis

In another prospective study by Sarin s group, consecutive patients with decompensated cirrhosis were randomly assigned to groups given subcutaneous G-CSF for 5 days and then every 3rd day (12 total doses), along with subcutaneous darbepoetin alpha (40 mcg/week) for 4 weeks or only placebos. All patients also received standard medical therapy and were followed for 12 months. Histology was performed on liver biopsies. The primary end point was survival at 12 months. A higher proportion of patients in the G-CSF group than controls survived until 12 months (68.6 *vs* 26.9%; p = 0.003). At 12 months, Child-Turcotte-Pugh (CTP) scores were reduced by 48.6% in the G-CSF group and 39.1% in the control group, from baseline (p = 0.001). Model for end-stage liver disease (MELD) scores were reduced by 40.4 and 33% respectively (p = 0.03). The need for large-volume paracentesis was significantly reduced in G-CSF group, compared with controls (p < 0.05). A lower proportion of patients in the G-CSF group developed septic shock (6.9%) during follow-up compared with controls (38.5%; p = 0.005). No major adverse events were observed in either group.^2^

There are also several studies in the literature exploring the role of G-CSF in acute on chronic liver failure (ACLF), which is a form of acute decompensation of liver cirrhosis. Garg et al^3^ observed that G-CSF mobilizes CD34+ cells and improved survival of patients with ACLF. The study showed G-CSF therapy leads to a significant increase in total leukocyte count across the total duration of therapy. The researchers observed G-CSF therapy also leads to a significant increase in the CD34 cell population in the liver tissue after 4 weeks of G-CSF administration. This proved the role of recruitment of BMC after G-CSF stimulation in patients with decompensated cirrhosis. An important observation of the study was that G-CSF therapy tends to prevent the development of multiorgan failure. This could be explained by prevention of sepsis in these patients with the drug. Neutrophil dysfunction has been shown to cause sepsis and further to the development of HRS and hepatic encephalopathy in patients with decompen-sated cirrhosis. The G-CSF therapy further showed an improvement of CTP and MELD scores and significantly improved survival in patients with ACLF, which is a variant of hepatic decompensation occurring following an acute insult in patients with previously diagnosed or undiagnosed cirrhosis of liver.

**Table 2:** **Table 2:** Demographic characteristics of study population

*Sex*		*Frequency*		*Percent*	
F		7		41.0	
M		10		59.0	
Total		17		100.0	

In their study, Duan et al^4^ observed that G-CSF therapy improves survival in patients with hepatitis B virus (HBV)-associated ACLF. The study showed G-CSF therapy increased peripheral neutrophil count and CD34+ cell count in patients with HBV-associated ACLF. Patients treated with G-CSF not only demonstrated a significantly better 90-day survival rate, CTP, and MELD scores, but were also less likely to develop HRS and sepsis compared with controls. This could be explained by increased numbers of neutrophils in the patients.^3^

Although our study did not reveal such encouraging findings as evidenced in the studies discussed here, it did establish the safety of G-CSF therapy in patients with decompensated cirrhosis of liver. This may also be associated with improved survival in such patients, as 88% patients included in the study were alive at the end of the follow-up period.

It is therefore hypothesized that a new study should be designed where stem cells will be harvested from the peripheral blood after stimulation of bone marrow with more frequent doses of G-CSF followed by autolo-gous transfusion of harvested stem cells, as that may result in much better outcome. The outcome can be even better if stem cell may be given in the liver by some prosthesis.
